# Bidesmosidic betulin saponin bearing L-rhamnopyranoside moieties induces apoptosis and inhibition of lung cancer cells growth *in vitro* and *in vivo*

**DOI:** 10.1371/journal.pone.0193386

**Published:** 2018-03-14

**Authors:** Mouadh Mihoub, André Pichette, Balla Sylla, Charles Gauthier, Jean Legault

**Affiliations:** 1 Chaire de Recherche sur les Agents Anticancéreux d’Origine Naturelle, Laboratoire LASEVE, Université du Québec à Chicoutimi, Département des Sciences Fondamentales, 555 boul. de l’Université, Chicoutimi (Québec), Canada; 2 INRS-Institut Armand-Frappier, Université du Québec, 531 boul. des Prairies, Laval (Québec), Canada; Institute of Biochemistry and Biotechnology, TAIWAN

## Abstract

Betulin has a wide range of biological and pharmacological properties with its anticancer activity attracting most of the attention as it offers a possible alternative treatment to chemotherapy. However, betulin’s *in vivo* biological effectiveness is limited by its poor solubility. As such, we synthesized polar glycosylated derivatives to increase its hydrosolubility and enhance its pharmacological properties. Among these synthesized compounds, 28-*O*-α-l-rhamnopyranosylbetulin 3β-*O*-α-l-rhamnopyranoside (Bi-L-RhamBet) was assessed for its cytotoxic effects against a suite of lung cancer cell lines. We also investigated its mechanism of action using an A549 lung cancer cell line. Our results showed that Bi-L-RhamBet exhibited potent cytotoxic activity toward lung cancer cell lines including A549, NCI-H2087, NCI-H522, NCI-H1993 NCI-H1755, and LLC1 having IC_50_ values ranging from 2.9 to 5.9 μM. Moreover, Bi-L-RhamBet (50 mg/kg) significantly inhibited tumor growth with a treatment-to-control ratio (T/C) of 0.54 and a tumor growth inhibition rate of 46% at day 18 (p < 0.05). Microscopic observations of A549 cells, double stained with acridine orange and ethidium bromide, showed apoptotic features. Bi-L-RhamBet induced activation of pro-apoptotic caspases 8, 9, and 3/7 as well as causing DNA fragmentation. Moreover, a marked increase in mitochondrial ROS (mROS) was coupled with a reduction of mitochondrial potential. Interestingly, the presence of mitochondrial electron transport chain (ETC) inhibitors, including rotenone, malonate, and antimycin A, reduced mROS production, and the activation of caspases suggesting that Bi-L-RhamBet disturbs the ETC. Finally, dichloroacetate, a pyruvate dehydrogenase kinase inhibitor potentiated the cytotoxicity of Bi-L-RhamBet against A549 cells. Taken together, these data suggest that Bi-L-RhamBet can induce apoptotic cell death via disturbance of mitochondrial electron transfer chain, reduced ROS production, and decreased membrane potential.

## Introduction

Cancer remains a major public health problem in industrial countries due to an aging population coupled with increased risk factors such as smoking, obesity/excessive weight, and physical inactivity [1]. In 2012 alone, GLOBOCAN estimated ca. 14.1 million new cancer cases and 8.2 million cancer-related deaths worldwide. In developed countries, lung cancer is the leading cause of death from cancer [2]. Despite the development of several new targeted treatments (bevacizumab, erlotinib, ramucirumab, nintedanib, nivolumab) against non-small cell lung cancer (NSCLC), the overall five-year survival rate has increased only slightly over the last decade from 15.7% to 17.4% [3]. Therefore, new approaches are urgently needed to improve lung cancer survivorship.

In 2014, Newman and Giddings reported that 67% of small molecule antitumor drugs can be ascribed to being either natural products or inspired by natural products [4]. Research on natural products has led to the development of several antitumor drugs in use against NSCLC including paclitaxel, docetaxel, and vinorelbine [5]. Marine organisms, microorganisms, and plants are potential sources of new, natural antitumor agents. Natural products present diverse chemical scaffolds and act through several mechanisms of action such as the induction of apoptosis, the inhibition of angiogenesis, the permeabilization of mitochondria, and the inhibition of enzymes required for cellular metabolism and signal transduction [5].

**Fig 1:**
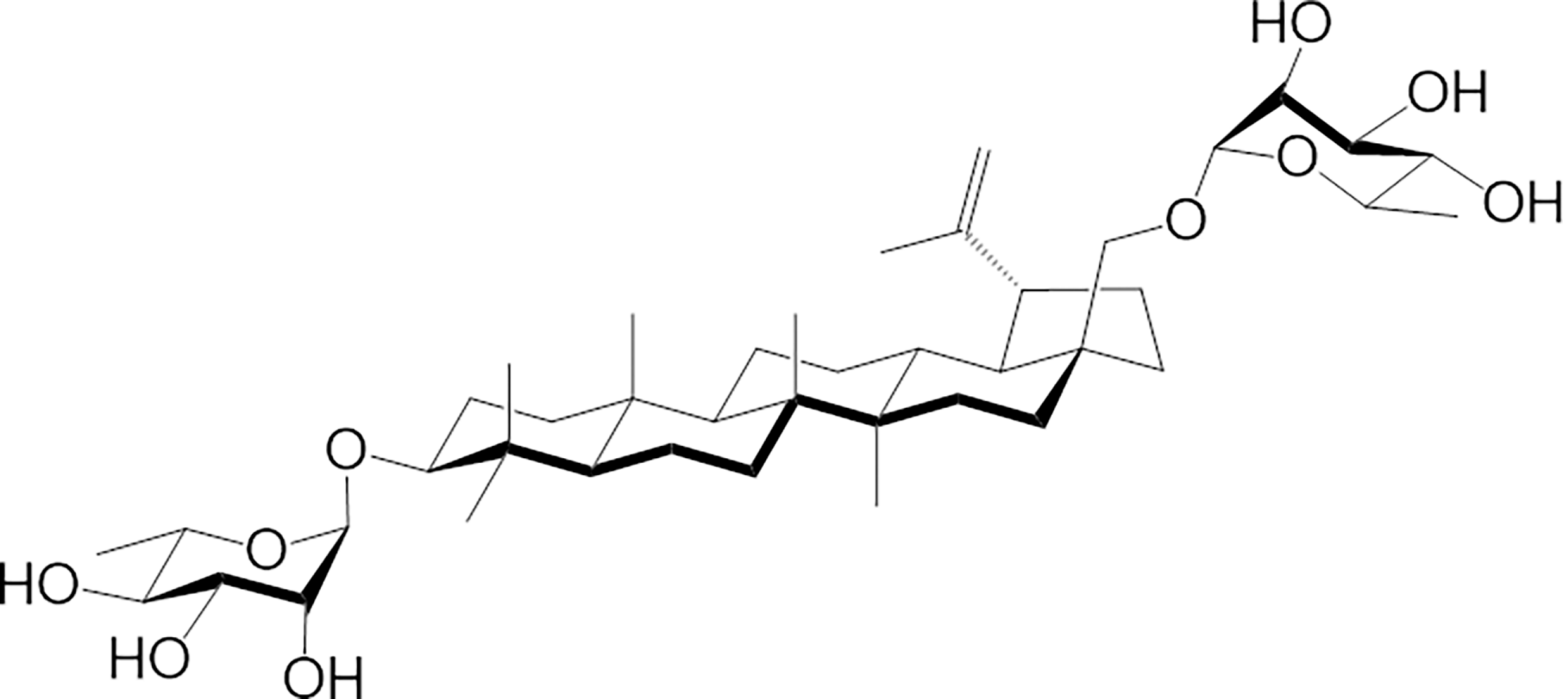
Molecular structure of 28-*O*-α-l-rhamnopyranosylbetulin 3β-*O*-α-l-rhamnopyranoside (Bi-L-RhamBet).

Betulin (lup-20(29)-ene-3β,28-diol) is a pentacyclic lupane-type triterpenoid that is found naturally in many plants, in particular in birch bark. Birch was often used by Native Americans for medicinal purposes [6]. We have previously observed the cytotoxicity and anti-proliferative actions of betulin against several human cancer cell lines including K562 (myelogenous leukemia), NB-1 (neuroblastoma) [7], TE671 (medulloblastoma), FTC 238 (thyroid carcinoma) [8], A549 (lung cancer), MCF-7 (breast adenocarcinoma), DLD-1 (colorectal adenocarcinoma) [9] [10], HepG2 (hepatoma) [11], MEL-2 (melanoma) [12], A431 (skin epidermoid carcinoma) [13], Jurkat E6.1 (T lymphoblast leukemia) [14], and EPG85 (gastric carcinoma) [15]. The cytotoxicity of betulin has been assessed using mouse melanoma and lymphoma cell lines having IC_50_ values lower than 14 μM [9] [16]. Betulin has also been tested against glioblastoma, cervical, and ovarian primary tumor cell cultures that had IC_50_ values ranging from 2.8 to 3.4 μM [14]. Betulin has a lipophilic structure and is, therefore, poorly soluble in water. This limits its pharmaceutical development [17]. Betulin has hydroxyl groups at both the C-3 and C-28 positions as well as a double C-C bonding at C-20. As such, different chemical modifications can be achieved at these sites to generate multiple derivatives [18]. In recent years, our research group has synthesized various *O*-glycosidic derivatives of betulin and betulinic acid via a stepwise glycosylation approach [9]. Among the synthesized saponins, 28-*O*-α-l-rhamnopyranosylbetulin 3β-*O*-α-l-rhamnopyranoside (Bi-L-RhamBet, see Fig 1) was found to be the most active against all tested cancer cell lines [10]. Moreover, Bi-L-RhamBet was not hemolytic, in contrast with monodesmoside derivatives, favoring its use as an anticancer agent [19].

In continuation with our previous investigations, the present study evaluates the cytotoxicity of Bi-L-RhamBet against a series of NSCLC cell lines and details the antitumoral activity in LLC1 tumor-bearing mice. The mechanisms of action were also investigated *in vitro* against human lung carcinoma cells, A549.

## Material and methods

### Materials

DMSO (dimethylsulfoxide) was purchased from Fisher Scientific. Staurosporine was purchased from LC Laboratories, while etoposide, phenol/chloroform/isoamyl alcohol mixture, and RNase A were purchased from Sigma-Aldrich® Canada. Ethidium bromide was purchased from Fluka. β-Hederin was purchased from ChemFaces. Acridine orange and Image-iT™ TMRM Reagent were purchased from Molecular Probes® Webinars, ThermoFisher Scientific. Caspase-Glo® 9, Caspase-Glo® 8, Caspase-Glo® 3/7 were purchased from Promega. Dihydrorhodamine 123 was purchased from Cayman Chemical. 28-*O*-α-l-Rhamnopyranosylbetulin 3β-*O*-α-l-rhamnopyranoside (Bi-L-RhamBet) was synthesized following protocols presented in our previous study [10]. Bi-L-RhamBet was further purified by preparative HPLC.

### Preparative HPLC

Preparative HPLC separation (Agilent 1100) was carried out on a 21.2 × 250 mm Eclipse prep-XDB- C18 column using a multiple wavelength detector and an automatic fraction collector. Chromatographic conditions were the following: gradient elution with H_2_O:CH_3_CN (90:10→0:100) at flow rate of 20 mL/min for 25 min, retention time: 17.13 min.

### Cell lines and culture conditions

Human lung carcinoma (A549) and human non-small cell lung adenocarcinoma cell lines at various stages (including NCI-H23, NCI-H2087 (stage 1), NCI-H522 (stage 2), NCI-H1993 (stage 3), NCI-H1755 (stage 4)) as well as human normal lung fibroblast cell lines MRC-5 and HEL299 were obtained from the American Type Culture Collection (ATCC). All cell lines were cultured in DMEM or RPMI-1640, supplemented by 10% fetal bovine serum (Hyclone), penicillin (100 IU/mL), and streptomycin (100 μg/mL). Cells were kept at 37°C in a humidified environment with 5% CO_2_.

### Cytotoxicity assay

Cancer and healthy cell line cytotoxicity was assessed by resazurin assay [20], as described previously [19], and confirmed by a Hoescht 33342 test [21]. To summarize, 96-well plates (Costar®, Corning Inc.) were seeded with cells (5×10^3^ per well) and treated with increasing concentrations of Bi-L-RhamBet in DMSO. After 48 h, the fluorescence was measured on an automated 96-well Fluoroskan Ascent FL^TM^ plate reader (Labsystems) using excitation and emission wavelengths of 530/590 and 350/461 nm, for the resazurin assay and the Hoescht, test, respectively. For the kinetic study, incubation was performed for 2, 4, 6, 8, 12, and 24 h periods. Each experiment was carried out in triplicate and the results are representative of at least three different experiments. The results are expressed as the concentration inhibiting fifty percent of cell growth (IC_50_).

### Antitumoral activity of Bi-L-RhamBet against LLC1 tumor-bearing mice

Male C57BL/6NCrl mice (36–40 days, 22 g, from Charles River Laboratories, Saint-Constant, Québec) were housed in pathogen-free conditions at 25–27°C and 50–70% humidity with a 12-h light/dark cycle. On day 0, Lewis lung carcinoma (LLC1) cells (1×10^6^) in 100 μM phosphate-buffered saline (PBS) were injected subcutaneously into the right flank of the mice. From day 1 to day 4, 100 μL of Bi-L-RhamBet (25 or 50 mg/kg) solubilized in 10% DMSO and 6% Tween 80 in PBS and vehicle were administered intravenously by injection into the tail vein of mice. Tumor growth was assessed from day 10 to day 18 using caliper measurements. The tumor volume (mm^3^) was estimated using the formula V = (W(2)×L)/2, where V is tumor volume, W is tumor width, and L is tumor length. All animal experimentation was approved by the Canadian Council on Animal Care (CCAC) and was undertaken at the Université du Québec à Chicoutimi (UQAC).

### Analysis of the cell cycle by flow cytometry

A549 cells were washed with PBS and fixed with 70% ice-cold ethanol. The cells were then resuspended in 0.5% Triton X-100, 1 mg/mL DNase-free RNase A, and 1 μg/mL propidium iodide (PI). Subsequently, cell suspensions were incubated in the dark for 30 min at 37°C and analyzed by Beckman Coulter Epics XL flow cytometer.

### Acridine orange/ethidium bromide staining

Apoptosis studies were performed with double staining assay using acridine orange (AO) and ethidium bromide (EB) [22]. AO (15 mg) and EB (50 mg) were dissolved in 1 mL of 95% ethanol and then added to 49 mL of PBS, gently mixed, aliquoted, and stocked at –20°C. Before use, the stock solution was diluted 1/10 in PBS (pH 7.4). Growing A549 cells (3×10^3^ cells for controls and 6×10^3^ cells for treatments) were plated onto a 96-well plate and incubated for 14 h. After treatment (treated with staurosporine (STS), β-hederin (Hed), or Bi-L-RhamBet), cells were re-incubated for 3 h. To estimate the apoptotic or necrotic effects of the compounds, supernatants were removed by aspiration and cells were washed with PBS, then incubated for 5 min with the dual fluorescent staining solution (AO/EB). Observations were performed with Cytation3 (cell imaging multimode reader) using excitation and emission wavelengths of 530 and 590 nm, respectively.

### Caspase assays

The effects of Bi-L-RhamBet, Hed, and STS on caspase (3/7, 8, and 9) activity was determined using Caspase-Glo (Promega). Treatments were also performed with Bi-L-RhamBet associated with 10 μM of antimycin A, 10 mM of malonate and 10 μM rotenone. Controls did not have added antimycin, malonate, or rotenone. Briefly, 96-wells plates were seeded with 2×10^4^ A549 cells per well and incubated for 14 h. After treatments with different compounds at periods of 2, 4, 6, 8, and 10 h, caspase substrate was added and plates were incubated in the dark for 1 h. Luminescence was measured by Cytation3.

### DNA fragmentation analysis

DNA fragmentation was analyzed as described by Wang et al. [23] with some minor modifications. A549 cells were harvested by centrifugation, dissolved in 300 μL of lysis buffer (50 mM Tris-HCl, pH 8.0, 10 mM EDTA, 2% SDS) and incubated for 6 h at 55°C with 200 μg/mL proteinase K. The lysate was then treated with 2 mg/mL RNase A and incubated at 55°C for 2 h. DNA was extracted with chloroform/phenol/isoamyl alcohol (24/25/1, v/v/v). For its precipitation, 1/10 v of 3 M sodium acetate pH 5.2 was added, followed by 2 v 100% alcohol. DNA (15 μg) was then transferred to 1.2% agarose gel and electrophoresis was carried out at 70 V.

### Mitochondrial reactive oxygen species (mROS) assay

The mROS was measured by the oxidation of DHR123 to rhodamine123 [24, 25]. Briefly, A549 cells, previously incubated in 96-well plates and treated with different cytotoxins, were incubated for 30 min with 10 mM DHR123 and then washed twice with PBS and observed under Cytation3. The excitation and emission wavelengths were 507 nm and 529 nm, respectively. Cells were pretreated for 30 min with 10 μM of antimycin A, 10 mM of malonate, and 10 μM rotenone. Controls did not receive this pretreatment.

### Mitochondrial membrane potential assay

A549 cells were incubated overnight in 96-well plates (10,000 cells per well). After 6 h of treatment with Bi-L-RhamBet, 25 nM of Image-iT™ TMRM Reagent (a mitochondrial membrane potential indicator) was added. Cells were incubated for 20 min and then washed twice with PBS and observed under Cytation3.

### Statistical analysis

Values are expressed as mean ± standard deviation at least three determinations. The results were analyzed by the Kruskal-Wallis One Way Test followed by post-hoc Student-Newman-Keuls’ test using SigmaStat 3.5 software. *P* ≤ 0.05 was considered as significantly different.

## Results and discussion

### Bi-L-RhamBet inhibits the *in vitro* and *in vivo* growth of lung cancer cells

**Fig 2:**
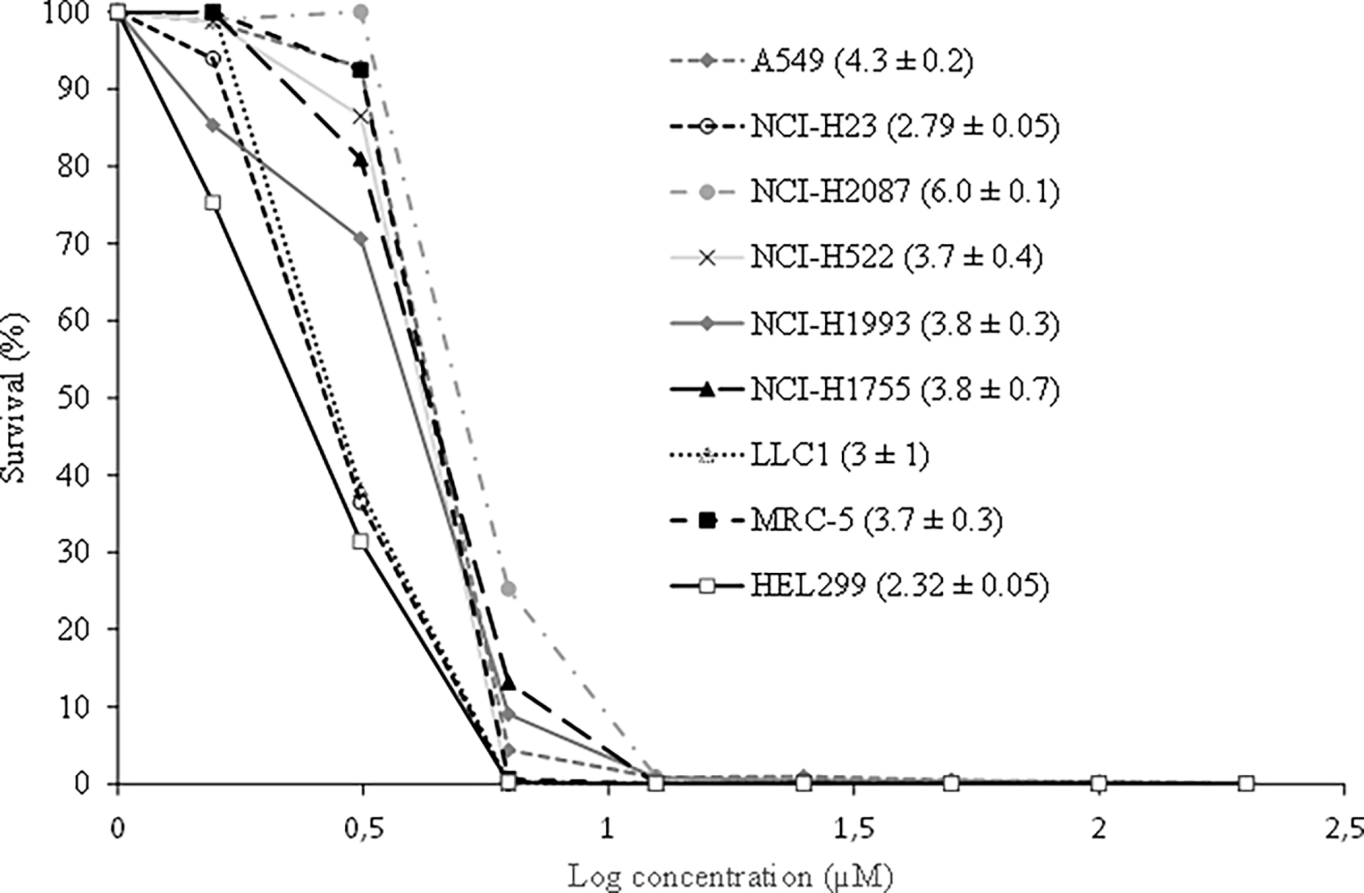
Bi-L-RhamBet induces growth inhibition of healthy and cancer cell lines. Survival of human healthy lung cell lines (MRC-5; HEL299), mouse Lewis lung cancer cells (LLC1), and human non-small cell lung cancer cells of different stages including: A549, NCI-H23, NCI-H2087 (stage 1), NCI-H522 (stage 2), NCI-H1993 (stage 3a), and NCI-H1755 (stage 4) all decrease with increased concentrations of Bi-L-RhamBet. The values in parentheses correspond to the concentrations inhibiting fifty percent of the cell growth (IC_50_). They represent mean values ± standard deviation of triplicates (n = 3) and are representative of three independent experiments.

All tested cell lines were incubated in the presence or absence of increasing concentrations of Bi-L-RhamBet that ranged from 1.5 to 25 μM. Our results show that Bi-L-RhamBet inhibited the survival of cancer cell lines with IC_50_ ranging from 2.8 to 5.9 μM (Fig 2). However, as previously observed [10], Bi-L-RhamBet was not selective against cancer cell lines when compared against the healthy cell lines of MRC-5 and HEL299 suggesting possible *in vivo* side effects. To investigate this possibility, the toxicity of Bi-L-RhamBet was assessed on C57BL/6NCrl mice. Bi-L-RhamBet at doses of 25, 50, and 75 mg/kg were administered intravenously in healthy mice. Interestingly, no toxicity was observed at doses of 25 and 50 mg/kg.

**Fig 3:**
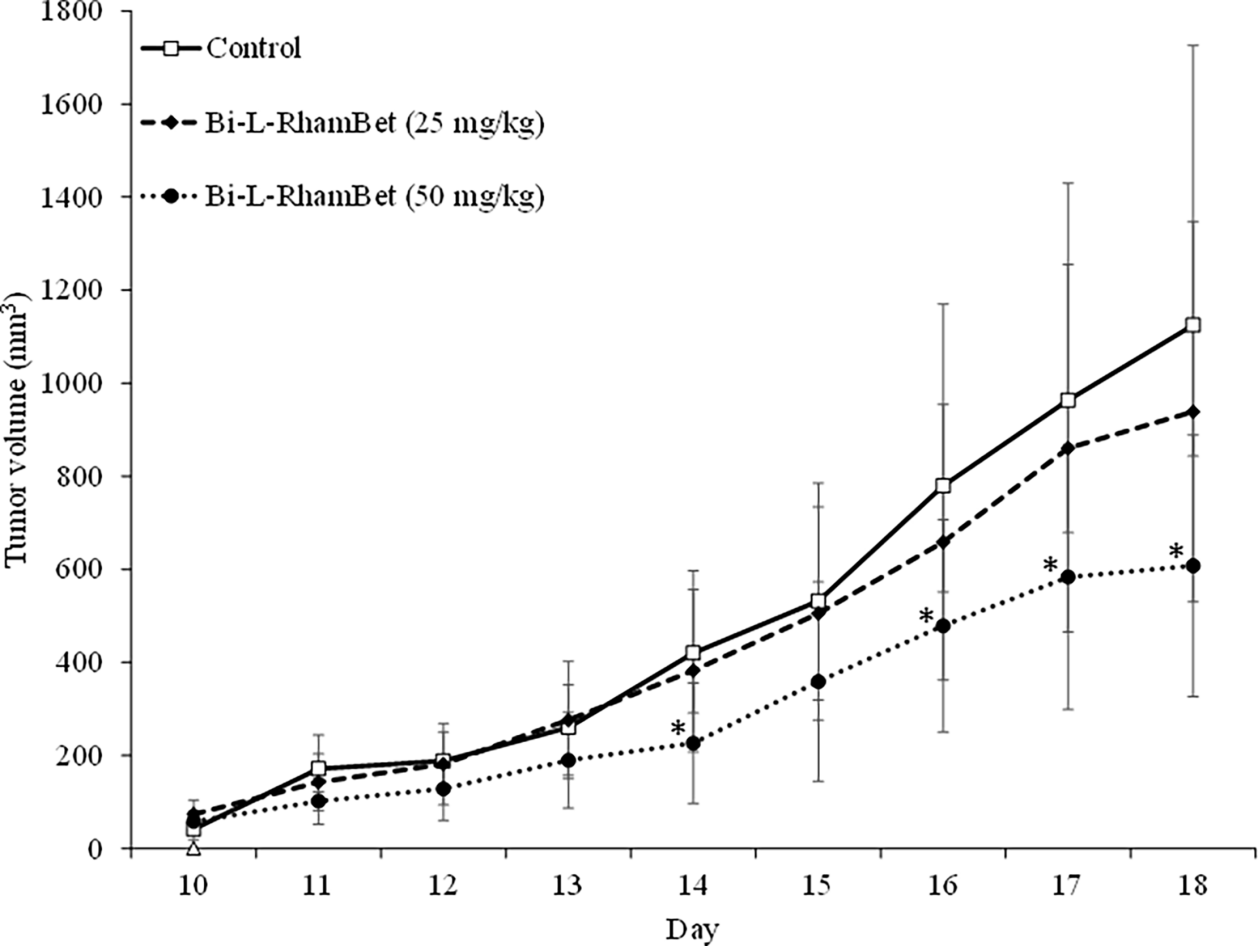
Tumor growth inhibition induced by Bi-L-RhamBet. Lewis lung tumor-bearing mice were untreated (control) or given doses of 25 or 50 mg/kg of Bi-L-RhamBet from days 1 to 4. The results are expressed as tumor volume in mm^3^ recorded between days 10 and 18. Data represent mean values ± standard deviation for ten mice (n = 10). *Values are significantly different from those of untreated (control) mice; Kruskal-Wallis One Way test followed by post-hoc Student-Newman-Keuls’ test.

**Fig 4:**
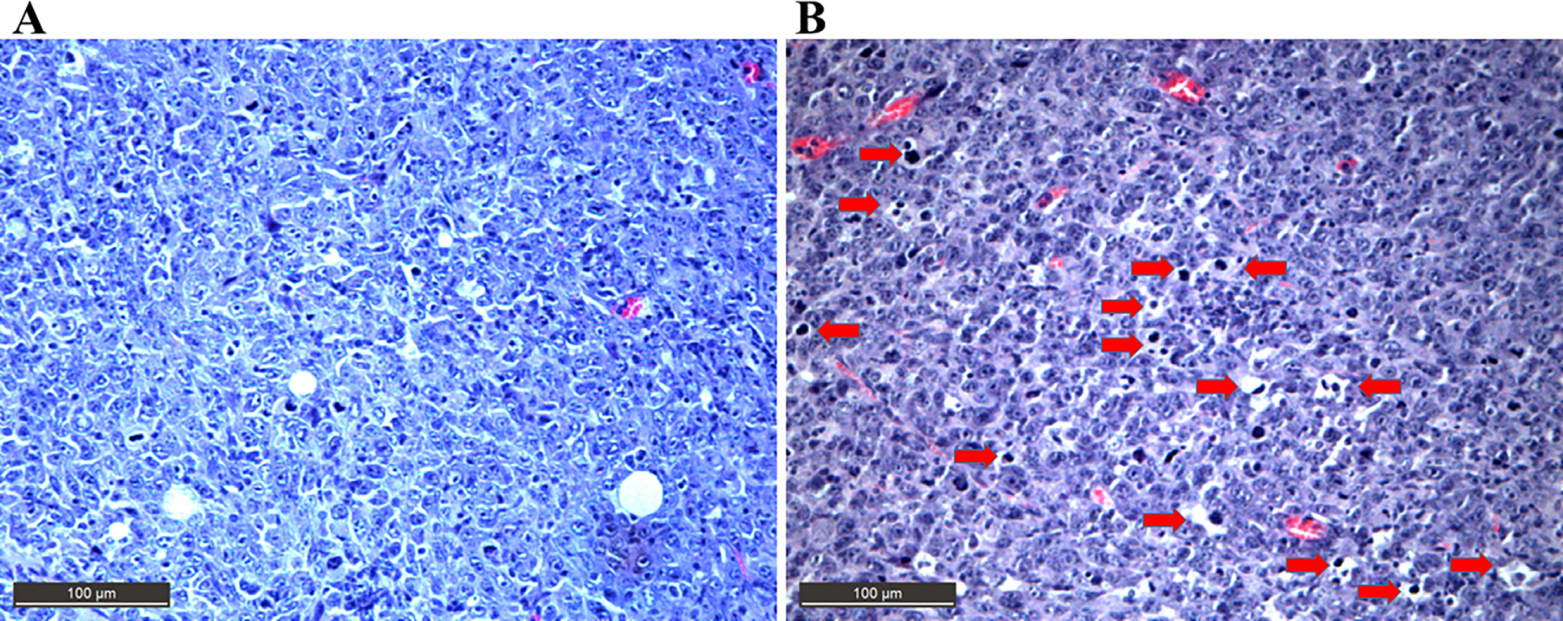
Hematoxylin and eosin-stained sections of Lewis lung tumor-bearing mice. Images of cells from the control (A) or treatment with 50 mg/kg of Bi-L-RhamBet (B). Red arrows indicate the presence of condensed chromatin (pyknosis) suggesting apoptotic cell death. Magnification at 400×. The section is representative of three different mice.

In addition, the antitumoral activity of Bi-L-RhamBet was evaluated on subcutaneous LLC1-bearing mice. On day 0, LLC1 cells were inoculated subcutaneously on the flank of the mice with 25 mg/kg Bi-L-RhamBet. The maximal tolerated dose of 50 mg/kg was then administered from day 1 to day 4. The tumor growth was measured from day 10 to day 18. The results show that Bi-L-RhamBet 50 mg/kg significantly inhibited tumor growth with a treatment-to-control ratio (T/C) ratio of 0.54 and a tumor growth inhibition rate (TGI) of 46% at day 18 (p <0.05) (Fig 3). The tumors were extracted, fixed, embedded in paraffin, and sliced prior to hematoxylin and eosin staining. In Fig 4, red arrows indicate the presence of condensed chromatin (pyknosis) suggesting cell death, possibly by apoptosis. The mechanism of action of Bi-L-RhamBet was then investigated *in vitro* using human lung carcinoma A549 cell lines.

### Bi-L-RhamBet blocks A549 cells in the G2/M phase of the cell cycle

**Fig 5:**
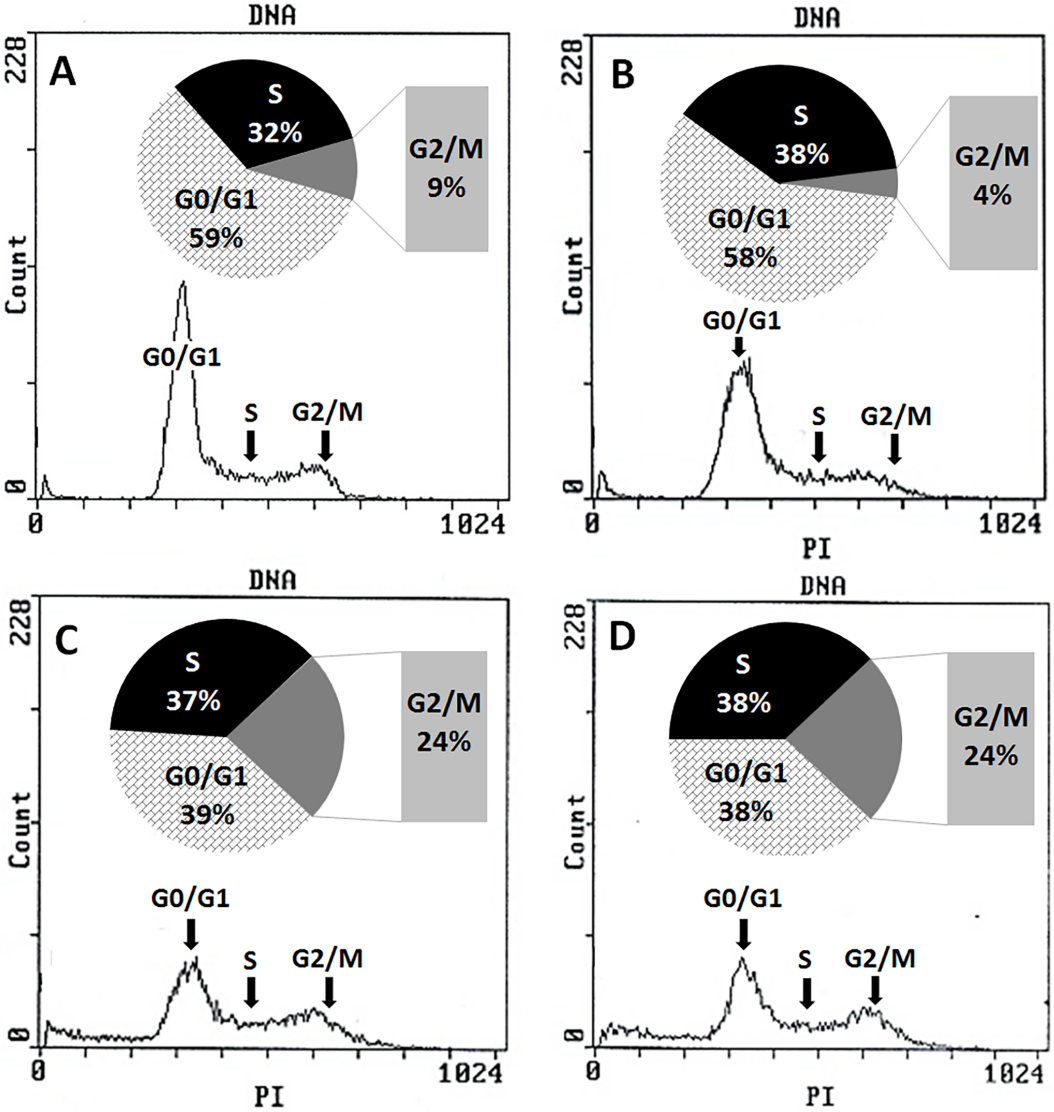
Effect of Bi-L-RhamBet on the cell cycle of A549 cancer cell lines. (A) untreated cells (control), A549 cells treated 24 h with 3.12 μM (B), 6.25 μM (C), and 12.5 μM (D) of Bi-L-RhamBet. DNA was stained with propidium iodide and analyzed by flow cytometry. This analysis is representative of three independent samples.

First, the effect of Bi-L-RhamBet was evaluated on the cellular cycle of A549 cells. Growing cells were treated (or not) over 24 h with 3.12, 6.25, and 12.5 μM of Bi-L-RhamBet. Cells were fixed and stained with PI and then analyzed by flow cytometry. The results showed that the distribution of control cells in each phase of cycle including G0/G1, S, and G2/M were 59%, 32%, and 9%, respectively (Fig 5A). Bi-L-RhamBet at concentrations of 6.25 and 12.5 μM induced a blockage in the G2/M with 24% of the cells in this phase after 24 h (Fig 5C and 5D). G2/M phase arrest is often associated with apoptosis [26]. Moreover, several triterpenoid saponins were found to block A549 cells in G2/M phase and induce apoptosis [27–30].

### Bi-L-RhamBet induces early morphological change and delays cytotoxicity related to apoptosis

**Fig 6:**
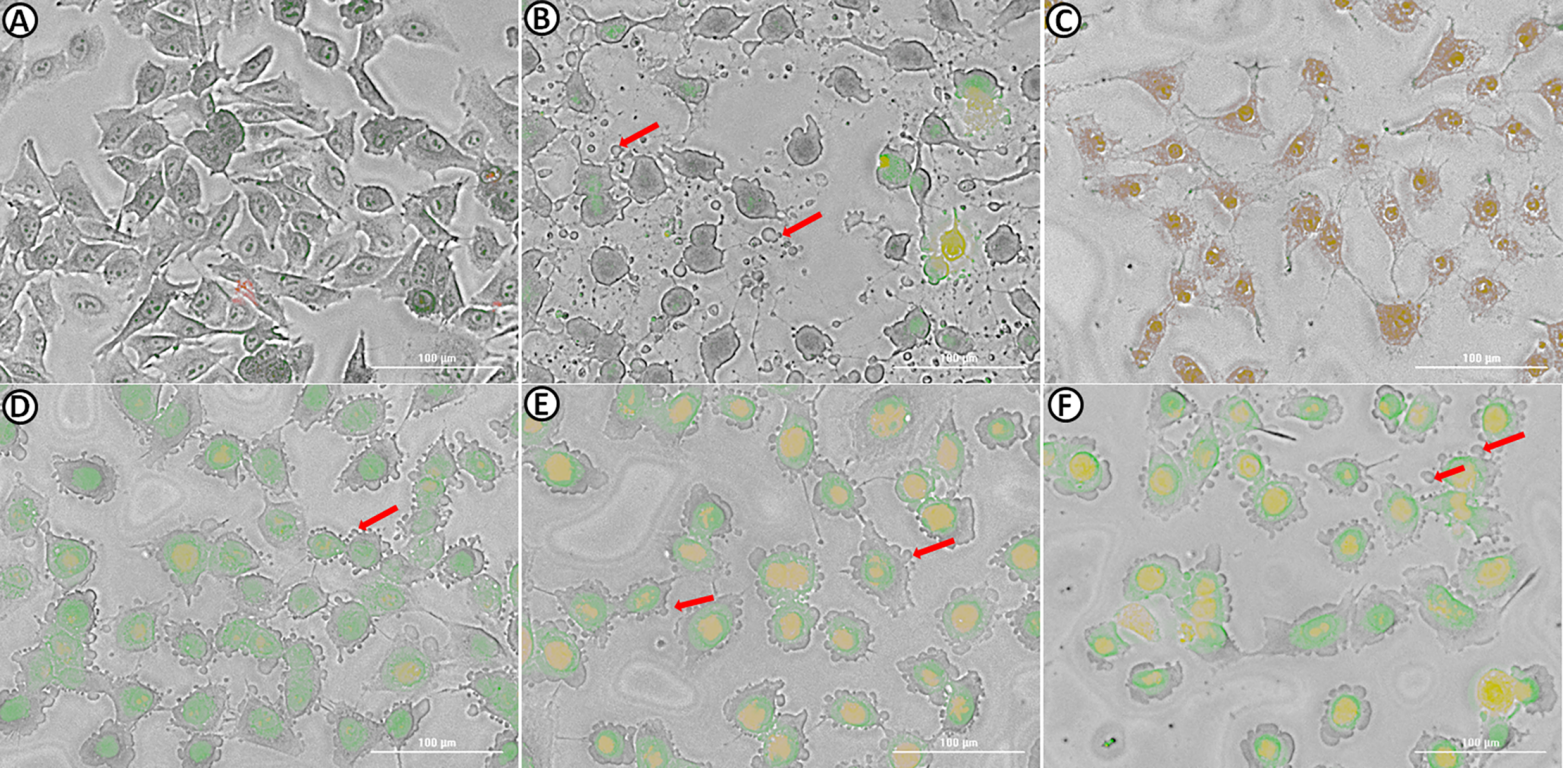
Morphological changes in A549 cells stained with acridine orange (AO) and ethidium bromide (EB). Cells were untreated (A) or treated for 3 h with 1 μM staurosporine (B), 10 μM β-hederin (C), 5 μM Bi-L-RhamBet (D), 10 μM Bi-L-RhamBet (E), and 20 μM Bi-L-RhamBet (F). A549 cells were stained with AO/EB for 5 min and then visualized using fluorescence microscopy. Arrows indicate apoptotic blebbing. This analysis is representative of three independent experiments.

Apoptosis is a programmed cell death regulated by activation of caspases induced by two main pathways: the death receptor (extrinsic pathway) or mitochondrial ROS (intrinsic pathway). Generally, this process occurs over several hours and is morphologically characterized by cell shrinkage and rounding, chromatin condensation, membrane blebbing, and the presence of apoptotic bodies. To investigate early morphological change induced by Bi-L-RhamBet, A549 cells were incubated for 3 h and stained with two dyes, AO and EB. In contrast to necrosis, the plasma membrane was not destroyed at first during apoptosis. Consequently, the membrane is permeable to AO but not to EB during early apoptosis. Staurosporine, a pro-apoptotic agent, and hemolytic β-hederin were used as controls to illustrate apoptotic and necrotic pathways, respectively [19] [31]. In contrast to β-hederin-treated cells, treatment with staurosporine clearly induced cell shrinkage, rounding, and apoptotic bodies (Fig 6), typical morphological changes in apoptotic cells [32]. As expected, cells treated with staurosporine were permeable to AO but not to EB while β-hederin was clearly permeable to EB showing a red-brown coloration indicating marked damage to the membrane. Moreover, cytotoxicity of β-hederin, determined by IC_50_ values (Table 1), was observed after only 2 h of treatment while the cytoxicity of staurosporine appeared after 12 h. Bi-L-RhamBet treated cells showed some characteristics of apoptotic morphological change including cell rounding and blebbing. Moreover, A549 cells treated with the lowest concentration of Bi-L-RhamBet (5 μM) were strongly marked in green indicating that cells were permeable to AO but relatively impermeable to EB. However, the membrane permeability to EB slightly increased at higher concentrations of Bi-L-RhamBet (10 and 20 μM) with a pronounced yellow color in the nucleus, as seen in later apoptotic appearances. Apoptosis can also increase the permeability of the plasma membrane to dyes but in a much less marked fashion than with hemolysis [33]. Similar to staurosporine, the cytotoxicity of was delayed with an IC_50_ of 45 μM after 12 h and 10.2 μM after 24 h (Table 1). Given these observations, Bi-L-RhamBet treatment seems to induce *in vitro* apoptotic cell death.

**Table 1:** Bi-L-RhamBet induces delayed cytotoxicity in A549 cells.

Compounds	Processing time					
	2 h	4 h	6 h	8 h	12 h	24 h
Bi-L-RhamBet	NA	NA	NA	NA	45 ± 8	10.2 ± 0.8
Staurosporine	NA	NA	NA	NA	4.3 ± 0.6	0.7 ± 0.2
β-Hederin	28 ± 2	20 ± 2	12.3 ± 0.8	18 ± 1	16.8 ± 0.7	14 ± 2
Etoposide^a^	NA	NA	NA	NA	NA	29 ± 4

Results are expressed as concentration inhibiting fifty percent of the cell growth (IC_50_).

NA: Not active; IC_50_ >50 μM was considered as being inactive.

Values are mean ± standard deviation of three replicates (n = 3).

^a^Positive control.

### Bi-L-RhamBet induces apoptotic caspases activation and DNA fragmentation

**Fig 7:**
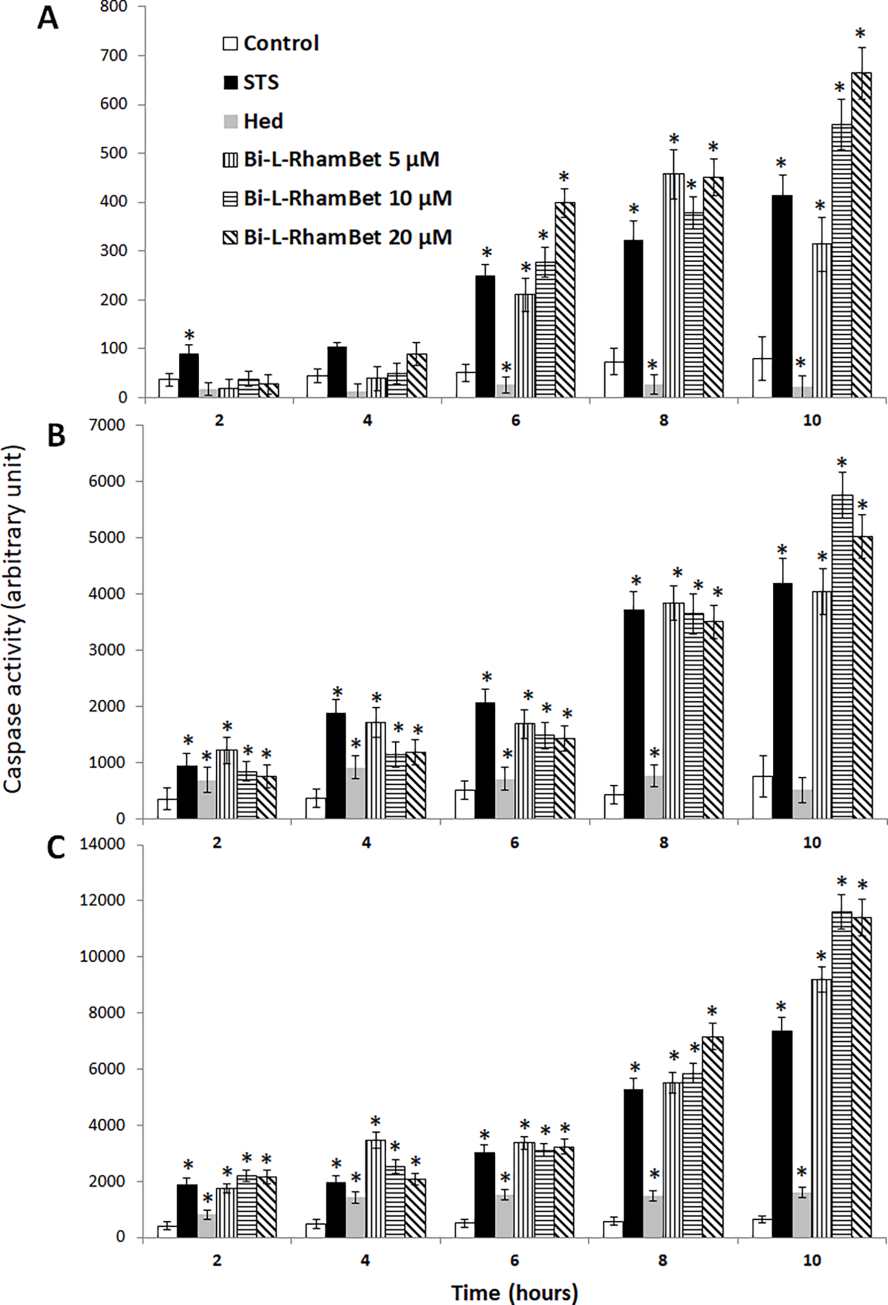
Kinetics of pro-apoptotic caspase activation induced by Bi-L-RhamBet. A549 cells were treated with 1 μM of Staurosporine (STS), 10 μM of x-hederin (Hed) and 5, 10, and 20 μM of Bi-L-RhamBet. The activity of caspase 8 (A), caspase 9 (B) and caspase 3/7 (C) was measured after 2, 4, 6, 8, and 10 h. *Values are significantly different from untreated (control) cells (n = 3); Kruskal-Wallis One Way test followed by post-hoc Student-Newman-Keuls’ test. This analysis is representative of three independent experiments.

Apoptotic cell death usually occurs through activation of caspases, a family of cysteine proteases [34]. Caspases cleave various cellular substrates producing many of the biochemical and morphological processes of apoptosis, such as membrane blebbing and DNA fragmentation [35]. The effect of Bi-L-RhamBet on caspase activation was assessed *in vitro* on A549 cells. Staurosporine and β-hederin were used as positive and negative controls, respectively. The cells were treated with staurosporine (1 μM), β-hederin (10 μM), and Bi-L-RhamBet (5, 10, 20 μM). The luminescence induced by the activation of caspases 8, 9, and 3/7 was measured after 2, 4, 6, 8, and 10 h. In contrast to β-hederin, staurosporine strongly activated caspases 8, 9, and 3/7 in a time-dependent manner (Fig 7). The Bi-L-RhamBet treated cells also activated caspases 8, 9, and 3/7 a in time-dependent manner until 8 h. Beyond 10 h of treatment, caspase activity of Bi-L-RhamBet at concentrations of 10 and 20 μM was significantly higher (p < 0.05) than for staurosporine-treated cells. Caspase 8 is activated when Fas ligand (FasL) or tumor necrosis factor-α (TNF-α) binds to a specific cell surface receptor (Fas and TNF, respectively). Caspase 9 is activated after the binding of cytochrome C and Apaf-1 to pro-caspase 9 following an increase of reactive oxygen species (ROS) and/or decrease of mitochondrial membrane potential. The activation of caspase 8 or 9 induces the activation of caspase effectors 3/7, which are implied in protein cleavages and DNA fragmentation [35]. DNA fragmentation is a key feature of apoptosis, initiated by the caspase-activated DNase [36].

**Fig 8:**
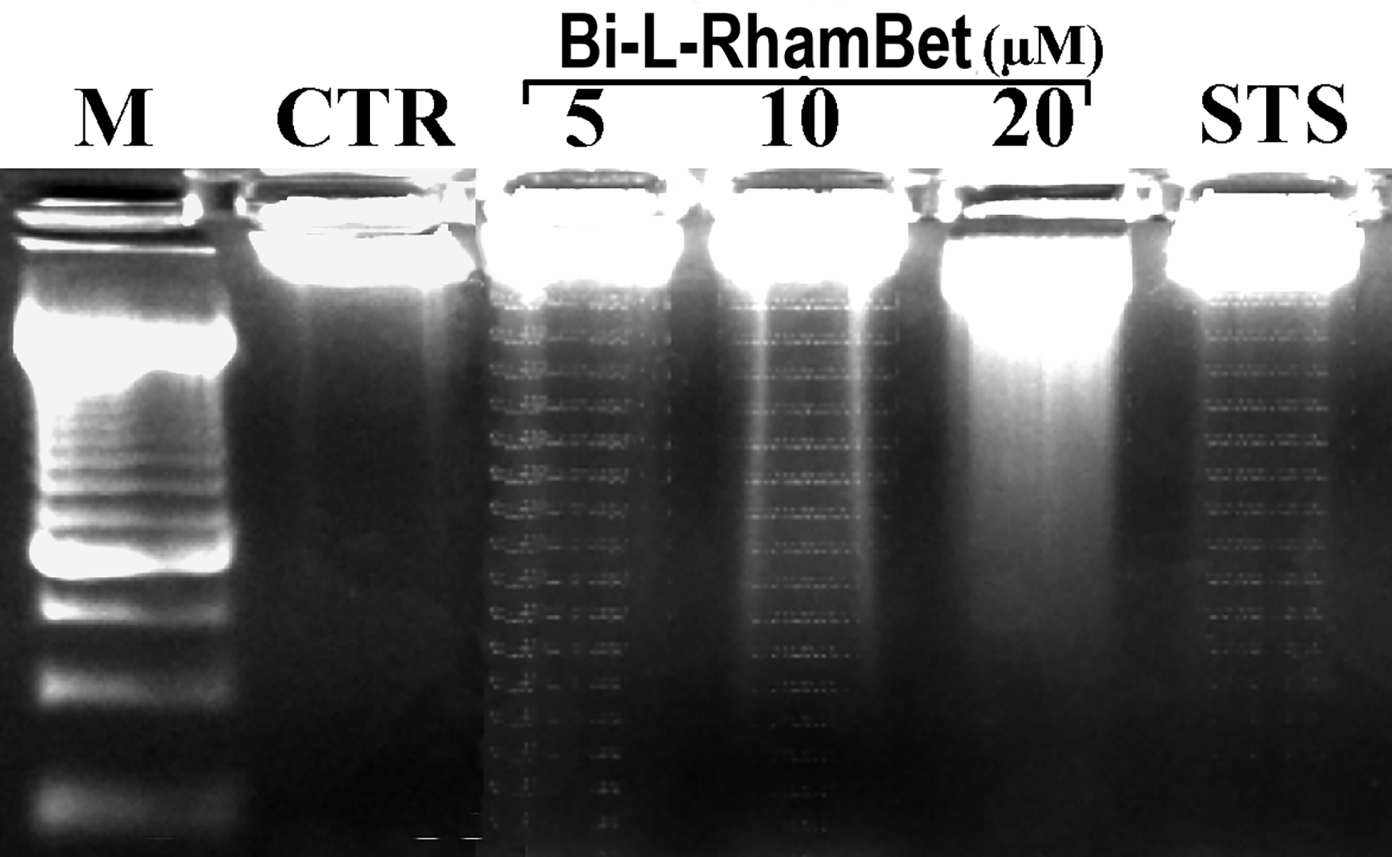
DNA electrophoretic profiles of treated A549 cells. Cells were either untreated (CTR) or treated for 24 h with either 5, 10, and 20 μM of Bi-L-RhamBet or 1 μM of staurosporine (STS). M: DNA size marker. This analysis is representative of three independent experiments.

To investigate the effect of Bi-L-RhamBet on this hallmark, the DNA of A549 cells treated with Bi-L-RhamBet was extracted and separated in 1.5% agarose gel. A degraded DNA profile was characteristic of apoptosis when cells were treated with 5 and 10 μM Bi-L-RhamBet during 24 h (Fig 8). Altogether, the results indicate that Bi-L-RhamBet induces cell death via both apoptotic pathways including a membrane death receptor pathway via caspase 8 and a mitochondrial pathway via caspase 9 followed by activation of caspase effectors 3/7 and DNA degradation.

### Bi-L-RhamBet induces caspase activation and apoptosis via mitochondrial electron transport chain disturbance and ROS production

**Fig 9:**
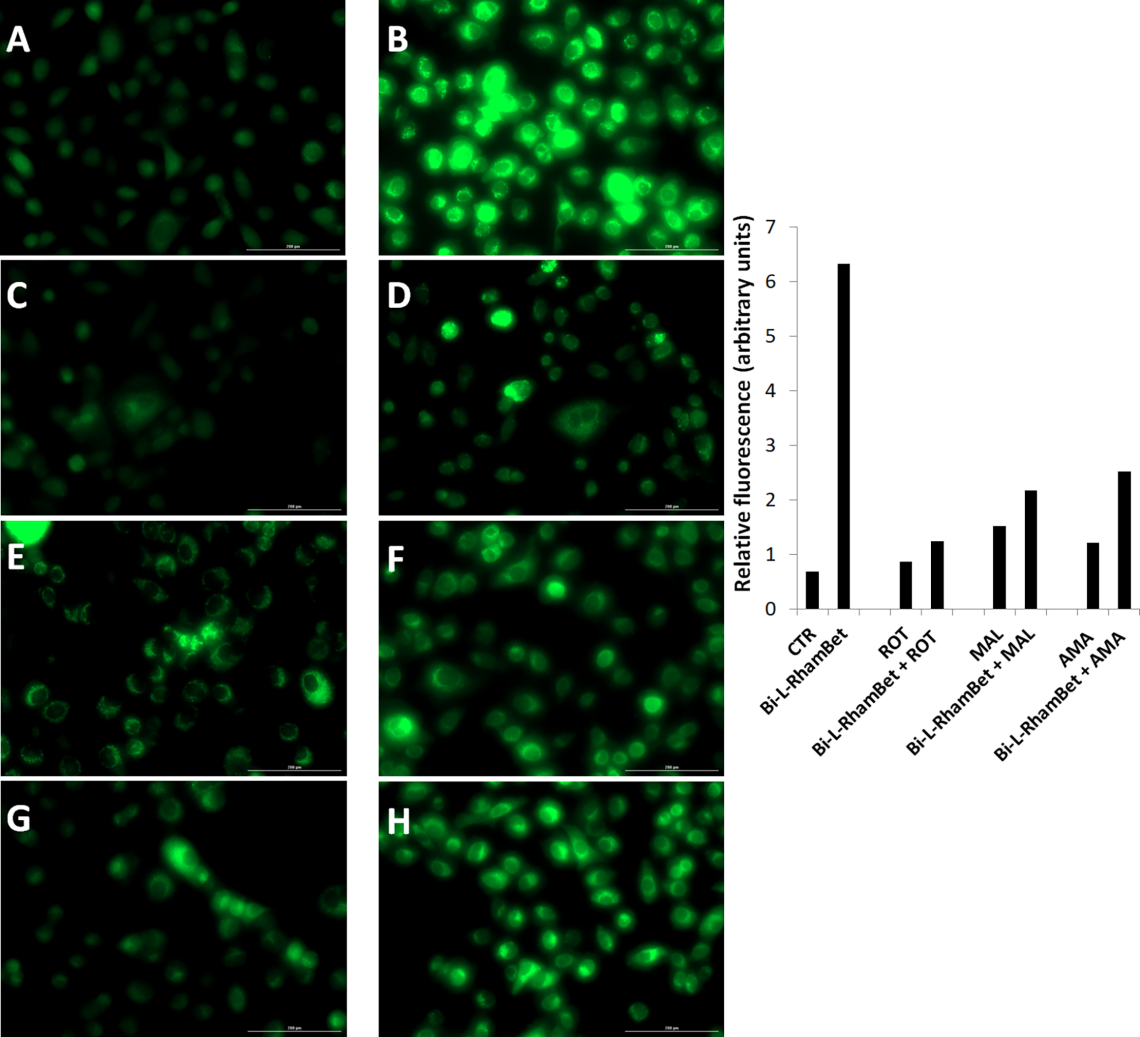
Mitochondrial ROS induced by Bi-L-RhamBet in A549 cells using DHR123. A549 cells were (A) untreated (CTR); or treated with 5 μM Bi-L-RhamBet (B), 10 μM rotenone (ROT) (C), 5 μM Bi-L-RhamBet + 10 μM ROT (D), 10 mM malonate (MAL) (E), 5 μM Bi-L-RhamBet + 10 mM MAL (F), 10 μM antimycin A (AMA) (G), and 5 μM Bi-L-RhamBet + 10 μM AMA (H). The fluorescence intensity of each image was quantified using Image J software and results are presented as histogram. This analysis is representative of three independent experiments.

**Fig 10:**
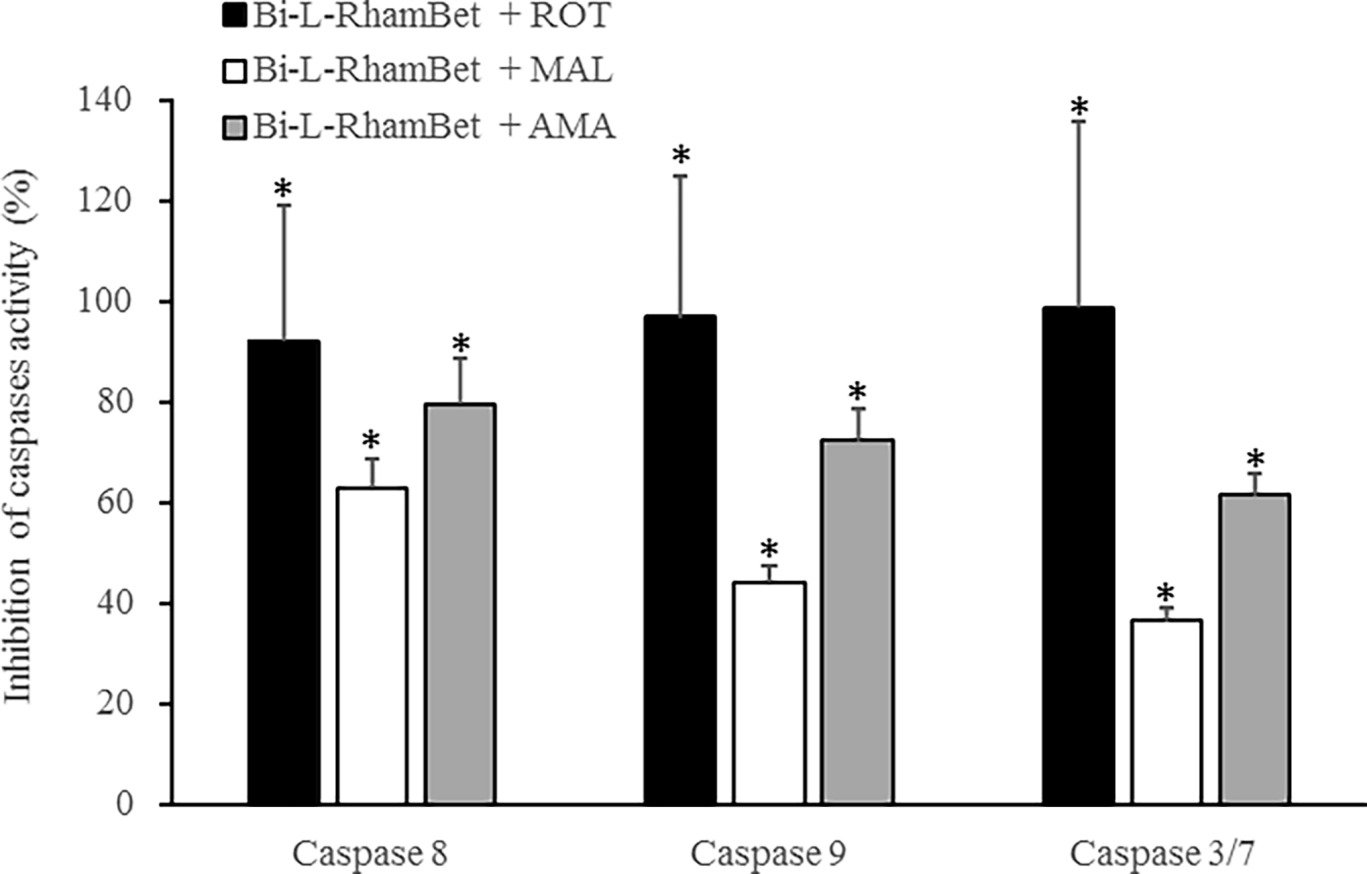
Mitochondrial chain transfer inhibitors prevent the activation of caspases induced by Bi-L-RhamBet in A549 cells. The cells were treated with 20 μM Bi-L-RhamBet and either 10 μM rotenone (ROT), 10 mM malonate (MAL), or 10 μM antimycine A (AMA). *Values are significantly different from Bi-L-RhamBet-only treated cells (n = 3); Kruskal-Wallis One Way test followed by post-hoc Student-Newman-Keuls’ test. This analysis is representative of three independent experiments.

**Fig 11:**
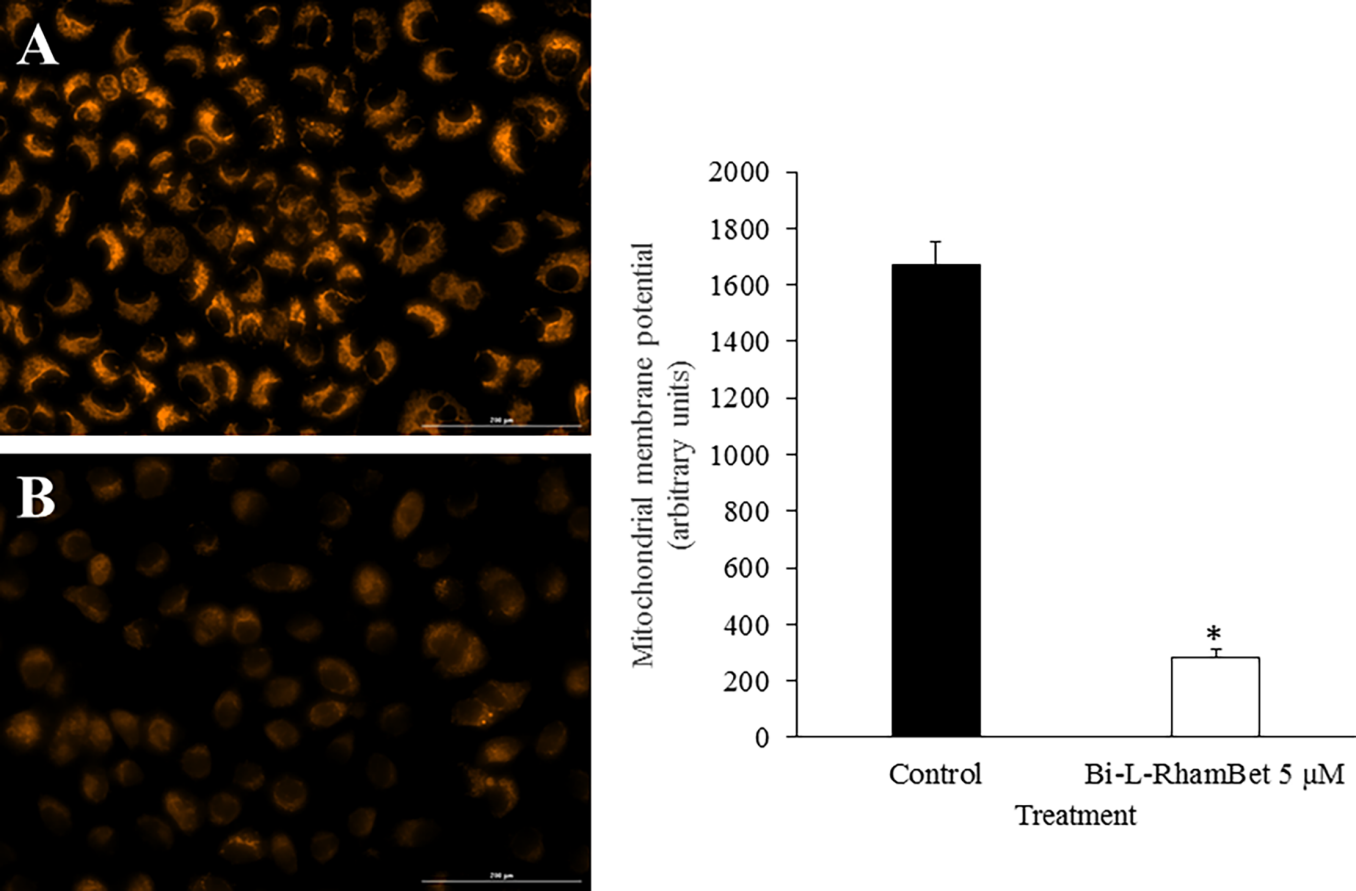
Mitochondrial membrane depolarization of A549 cells induced by Bi-L-RhamBet. A549 cells were (A) untreated (control) or (B) treated for 6 h with 5 μM of Bi-L-RhamBet. Mitochondrial membrane depolarization of A549 cells was assessed using the fluorescent dye TMRM. The intensity of TMRM fluorescence is proportional to the mitochondrial membrane potential. The fluorescence intensity was quantified using Image J software. *Values are significantly different from control (n = 3); Kruskal-Wallis One Way test followed by post-hoc Student-Newman-Keuls’ test. This analysis is representative of three independent experiments.

As previously mentioned, mitochondrial ROS (mROS) are implicated in the induction of apoptotic caspases. We evaluated the effects of Bi-L-RhamBet on the induction of mROS using dihydrorhodamine-123 (DHR123), a specific probe to detect ROS within mitochondria. A549 cells were treated with 5 μM of Bi-L-RhamBet in the presence of DHR123. In comparison with untreated cells, mROS increased by 8× after 6 h of treatment with Bi-L-RhamBet (Fig 9). A 30-min pretreatment with mitochondrial electron transport chain (ETC) inhibitors of complex I (rotenone), II (malonate), and III (antimycin A) decreased mROS production generated by Bi-L-RhamBet. We also evaluated the effect of ETC inhibitors on the induction of caspases by Bi-L-RhamBet. Rotenone, malonate, and antimycin A all inhibited the activation of caspases 8, 9 and 3/7 (Fig 10). Interestingly, rotenone inhibited 100% of caspase activities induced by Bi-L-RhamBet. Furthermore, evaluation of the mitochondrial potential by fluorescent labeling (Image-iT ™ Reagent TMRM) showed that a 6-h treatment of A549 cells with 5 μM Bi-L-RhamBet drastically reduced the mitochondrial potential of cells (Fig 11). Altogether, these results suggest that Bi-L-RhamBet disturbs mitochondrial ETC generating ROS, which reduces mitochondrial potential, activates caspases, and leads to apoptotic cell death.

### Dichloroacetate, a pyruvate dehydrogenase kinase inhibitor, potentiates Bi-RhamBet cytotoxicity in vitro

**Fig 12:**
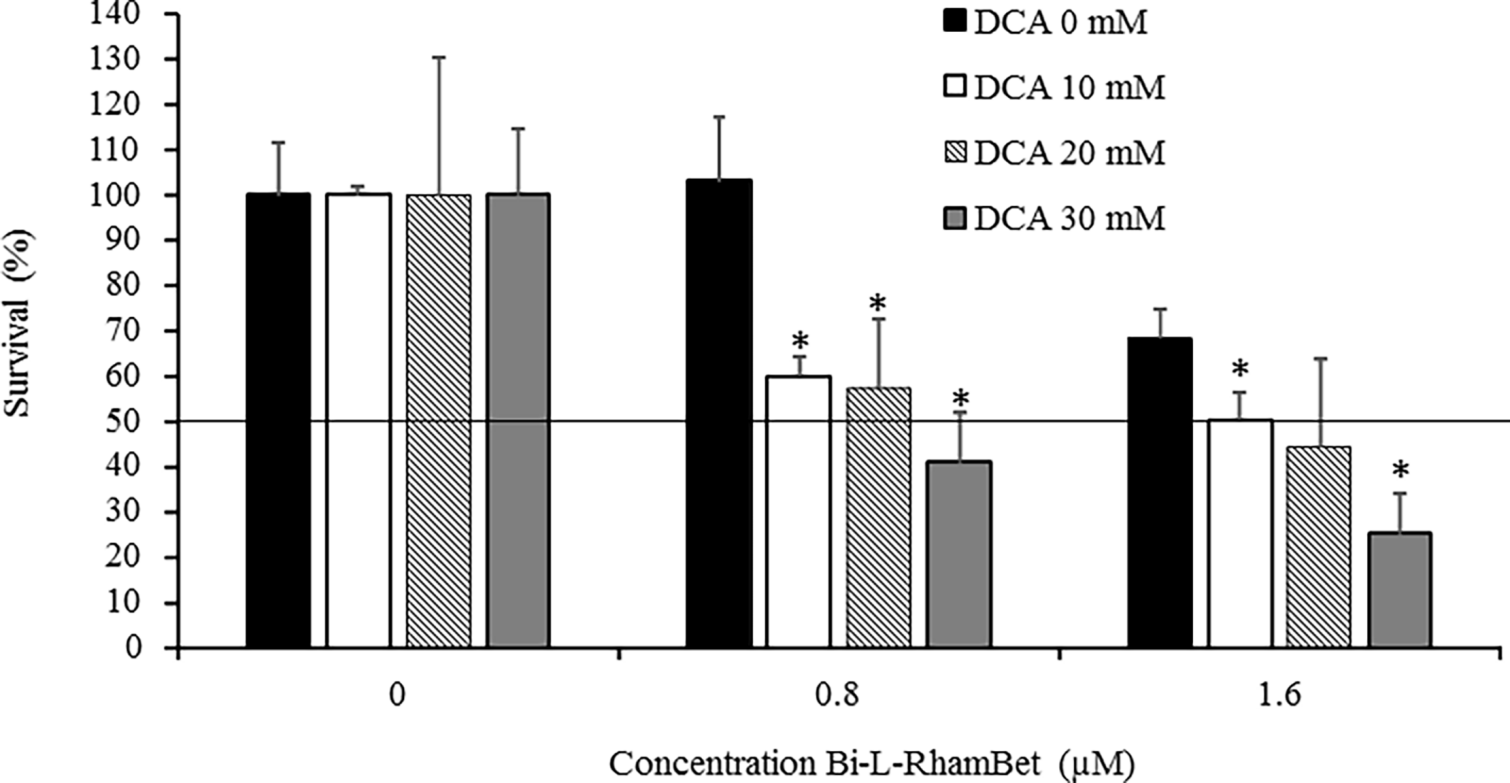
Dichloroacetate (DCA) potentiates the cytotoxicity of Bi-L-RhamBet in A549 cells. The cells were treated with 0.8 or1.6 μM of Bi-L-RhamBet combined with cytotoxic concentrations of 10, 20, and 30 mM of DCA. *Values are significantly different from Bi-L-RhamBet-only (0 mM of DCA) samples (n = 3); Kruskal-Wallis One Way test followed by post-hoc Student-Newman-Keuls’ test. This analysis is representative of three independent experiments.

Mitochondria is a vital organelle involved in producing cell energy via the Krebs cycle coupled with ETC. In cancer, alteration of mitochondrial metabolism promotes the progression of malignant tumors. Therefore, mitochondria are potential targets for chemotherapeutic agents [37]. For example, metformin, a mitochondrial complex I inhibitor, is currently undergoing clinical trials for cancer prevention and therapy [38]. Some tumors, on the other hand, use aerobic glycolysis to supply energy. This process, named the Warburg effect, can be reversed by dichloroacetate (DCA). Indeed, DCA can inhibit pyruvate dehydrogenase kinase that increases pyruvate dehydrogenase activity and the total oxidation of glucose in mitochondria via the Krebs cycle and ETC [39]. Consequently, the increased ETC activity induced by DCA could potentiate the cytotoxicity of Bi-L-RhamBet. We evaluated, *in vitro*, the potentiating activity of DCA combined with Bi-L-RhamBet. Non-cytotoxic concentrations of 0, 10, 20, and 30 mM of DCA were combined with 0, 0.8, and 1.6 μM of Bi-L-RhamBet in A549 cells for a period of 48 h. The results showed that 0.8 μM of Bi-L-RhamBet was not cytotoxic in comparison with untreated cells that had 100% survival rates (Fig 12). Interestingly, the combination of non-cytotoxic concentrations of DCA (10, 20, 30 mM) and Bi-L-RhamBet (0.8 μM) significantly inhibited cancer cell growth with inhibition ranging from 41% to 59%. The combination of 1.6 μM Bi-L-RhamBet with 30 mM DCA inhibited 75% of cell growth compared to only 30% when Bi-L-RhamBet was used alone. Altogether, this suggests that DCA potentiates the cytotoxicity of Bi-L-RhamBet.

## Conclusion

Our results show that Bi-L-RhamBet targets the mitochondria of lung cancer cells and disrupts the electron transfer chain. This results in the production of excess ROS and induces a reduction of the membrane potential. Subsequently, pro-apoptotic caspases are activated to induce the programmed cell death of cancer cells both *in vitro* and *in vivo*. Interestingly, dichloroacetate, a pyruvate dehydrogenase kinase inhibitor, potentiates the cytotoxicity of Bi-L-RhamBet *in vitro*. This suggests that their combined effect could be efficient in the *in vivo* treatment of lung tumors.
